# Assessment of correlation between conventional anthropometric and imaging-derived measures of body fat composition: a systematic literature review and meta-analysis of observational studies

**DOI:** 10.1186/s12880-023-01063-w

**Published:** 2023-09-14

**Authors:** Sofia Mouchti, Josefina Orliacq, Gillian Reeves, Zhengming Chen

**Affiliations:** 1 Cancer Epidemiology Unit, Richard Doll Building, Nuffield Department of Population Health, University of Oxford, Oxford, UK; 2 MRC Population Health Research Unit, Nuffield Department of Population Health, University of Oxford, Big Data Institute, Old Road Campus, Oxford, OX3 7LF UK

**Keywords:** Adiposity, Anthropometric, Imaging, MRI, DXA, Correlation, meta-analysis

## Abstract

**Background:**

In studies of the association of adiposity with disease risk, widely used anthropometric measures of adiposity (e.g. body-mass-index [BMI], waist circumference [WC], waist-hip ratio [WHR]) are simple and inexpensive to implement at scale. In contrast, imaging-based techniques (e.g. magnetic resonance imaging [MRI] and dual x-ray absorptiometry [DXA]) are expensive and labour intensive, but can provide more accurate quantification of body fat composition. There is, however, limited evidence about the relationship between conventional and imaging-derived measures of adiposity.

**Methods:**

We searched Scopus and Web of Science for published reports in English of conventional versus imaging-derived measurements of adiposity. We identified 42 articles (MRI = 22; DXA = 20) that met selection criteria, involving 42,556 (MRI = 15,130; DXA = 27,426) individuals recruited from community or hospital settings. Study-specific correlation coefficients (r) were transformed using Fisher’s Z transformation, and meta-analysed to yield weighted average correlations, both overall and by ancestry, sex and age, where feasible. Publication bias was investigated using funnel plots and Egger’s test.

**Results:**

Overall, 98% of participants were 18 + years old, 85% male and 95% White. BMI and WC were most strongly correlated with imaging-derived total abdominal (MRI-derived: r = 0.88-; DXA-derived: 0.50–0.86) and subcutaneous abdominal fat (MRI-derived: 0.83–0.85), but were less strongly correlated with visceral abdominal fat (MRI-derived: 0.76-0.79; DXA-derived: 0.80) and with DXA-derived %body fat (0.76). WHR was, at best, strongly correlated with imaging-derived total abdominal (MRI-derived: 0.60; DXA-derived: 0.13), and visceral abdominal fat (MRI-derived: 0.67; DXA-derived: 0.65), and moderately with subcutaneous abdominal (MRI-derived: 0.54), and with DXA-derived %body fat (0.58). All conventional adiposity measures were at best moderately correlated with hepatic fat (MRI-derived: 0.36–0.43). In general, correlations were stronger in women than in men, in Whites than in non-Whites, and in those aged 18 + years.

**Conclusions:**

In this meta-analysis, BMI and WC, but not WHR, were very strongly correlated with imaging-derived total and subcutaneous abdominal fat. By comparison, all three measures were moderately or strongly correlated with imaging-based visceral abdominal fat, with WC showing the greatest correlation. No anthropometric measure was substantially correlated with hepatic fat. Further larger studies are needed to compare these measures within the same study population, and to assess their relevance for disease risks in diverse populations.

**Supplementary Information:**

The online version contains supplementary material available at 10.1186/s12880-023-01063-w.

## Introduction

Globally, obesity affects about 700 million adults and the prevalence continues to rise steadily in most countries [1]. Higher levels of adiposity, which can be measured in various ways, are associated with impaired glucose and insulin resistance [2–4], hypertension [2, 4–9], and dyslipidaemia [2, 4, 10], and with increased risks of many different diseases such as cardiovascular disease (CVD) [11–14], diabetes [2, 4], and certain cancers (e.g. colon [15], breast [16] and prostate [17]). As a simple conventional anthropometric measurement, body mass index (BMI), defined as weight (kg) divided by height square (meter), has been widely used to measure body fat and to predict risks of morbidity [18] and mortality [1, 19]. However, people with similar BMI may have different comorbidities and disease risks [20], reflecting in part the inability of BMI to reliably measure total body fat, and the large variation of visceral fat distribution between individuals [21, 22]. Other conventional anthropometric measures include waist circumference (WC) and waist to hip ratio (WHR), which are often used as proxy measures of abdominal fat [23], and have been shown to be better predictors of certain diseases (e.g. type 2 diabetes) compared to BMI in some populations [24, 25]. There is, however, relatively little evidence as to how well these anthropometric measures of abdominal adiposity are likely to reflect the relative distribution of visceral as opposed to subcutaneous adipose tissue in the abdomen.

Adipose tissue exists under the skin (i.e. subcutaneous adipose tissue [SAT]), and around the muscles of the upper arm, buttocks, abdomen, hips and thighs. It also accumulates inside the peritoneal cavity and between the internal organs and torso (i.e. visceral adipose tissue ([VAT]). Moreover, it may be stored within tissues that do not normally store fat, such as the liver and the muscles, in which case it is termed ectopic fat [26]. The amount and distribution of adipose tissue among individuals differs by sex, age and ancestry but is also affected by many other factors (e.g. lifestyles, genetics). Adipose tissue is dynamically regulated, through the size and number of adipocytes, in response to varying energy demands [27]. A positive energy balance between intake and expenditure results in more fat storage and leads to weight gain.

Advances in imaging techniques have allowed more accurate quantification of body fat composition including visceral and ectopic fat deposition (e.g. cardiac and hepatic fat). Currently, magnetic resonance imaging (MRI) and dual X-ray absorptiometry imaging (DXA) are the two most commonly used imaging techniques. MRI involves three-dimensional imaging, enabling precise measurement and quantification of adipose tissue in all organs such as muscle, bone and regional areas including hepatic fat. However, it is more expensive, time consuming, and labour intensive compared with DXA [28]. By differentiating lean from fat tissue reliably, DXA provides good quantification of total and abdominal fat, but can only indirectly measure visceral fat by subtracting subcutaneous from the total abdominal fat. Nevertheless, compared with conventional anthropometric measures such as BMI and WC, these imaging-based techniques are difficult to implement at scale. Moreover, substantial uncertainty remains about the relationship between imaging-derived and conventional measures of adiposity and about their relevance, both qualitatively and quantitatively, for risks of specific diseases in diverse populations. Hence there is a need to bring together and review findings from all published studies which have assessed the agreement between imaging- and anthropometric- based measures of body fat.

We present a systematic review and meta-analyses of the published findings comparing conventional anthropometric with MRI- and DXA-based measures of body fat composition.

## Methods

We carried out a systematic literature review using the Preferred Reporting Items for Systematic Reviews and Meta-analyses (PRISMA) checklist [29].

### Eligibility criteria

Studies were eligible for inclusion if they reported correlations between MRI/DXA and any of the conventional anthropometric measures of body composition (e.g. BMI, WC, WHR) in adolescent or adult participants. Explicit details on inclusion and exclusion criteria can be found in Additional file (1) Two online biographic databases (Scopus and Web of Science) were searched covering a period from 1 to 2000 to 4 January 2023. Details of the search strategy are given in Additional file (2) Results were limited to publications in English. Although fat and adipose tissue have separate biochemical and metabolic characteristics, these terms have been used interchangeably for the purposes of the current study.

For included studies, two reviewers independently extracted the relevant pre-defined data regarding study population, participant demographic characteristics (e.g. sex, age, and ancestry), method of assessing weight, height and BMI, and adjustment for confounders. The measures of adiposity are composed of: (i) conventional anthropometric measures: BMI, WC, WHR; (ii) MRI-derived measures: abdominal total adipose tissue (ATAT), abdominal subcutaneous adipose tissue (ASAT), visceral adipose tissue (VAT), and hepatic fat; (iii) DXA-derived: ATAT, VAT, and %body fat (%BF) (defined as the percentage of total body fat mass over total body mass). The extracted information was recorded onto a spreadsheet and compared between two reviewers. Any inconsistencies were checked, reviewed and corrected upon discussion.

### Statistical analyses

We used the metacor function from the meta package in R to calculate the weighted average correlations between imaging- and anthropometric-based adiposity measures. In this method, study specific correlations (r) are transformed to Fisher’s Z values, with an estimated variance of 1/(n-3), where n is the number of participants included in the study. The overall weighted correlation for all studies was derived by applying study-specific weights proportional to the inverse of the variance of the study-specific Fisher’s Z values [30]. We defined absolute correlations of magnitude < 0.20 as very weak, 0.20–0.39 as weak, 0.40–0.59 as moderate, 0.6–0.79 as strong, and ≥ 0.8 as very strong. Heterogeneity in estimated correlations according to study, ancestry, sex and age was assessed using the Q-test [31]. Statistical tests with a p-value less or equal to 0.05 were considered significant. One included study [32] was extremely large compared with all other included studies and therefore contributed substantially to the overall findings [32]. To assess the potential impact of this study on the overall findings, we presented the results of the meta-analysis separately with and without inclusion of this study.

Publication bias was assessed using Funnel plots and Egger’s test [33]. All analyses were performed in programming language R version 4.1.1.

## Results

**Fig. 1:**
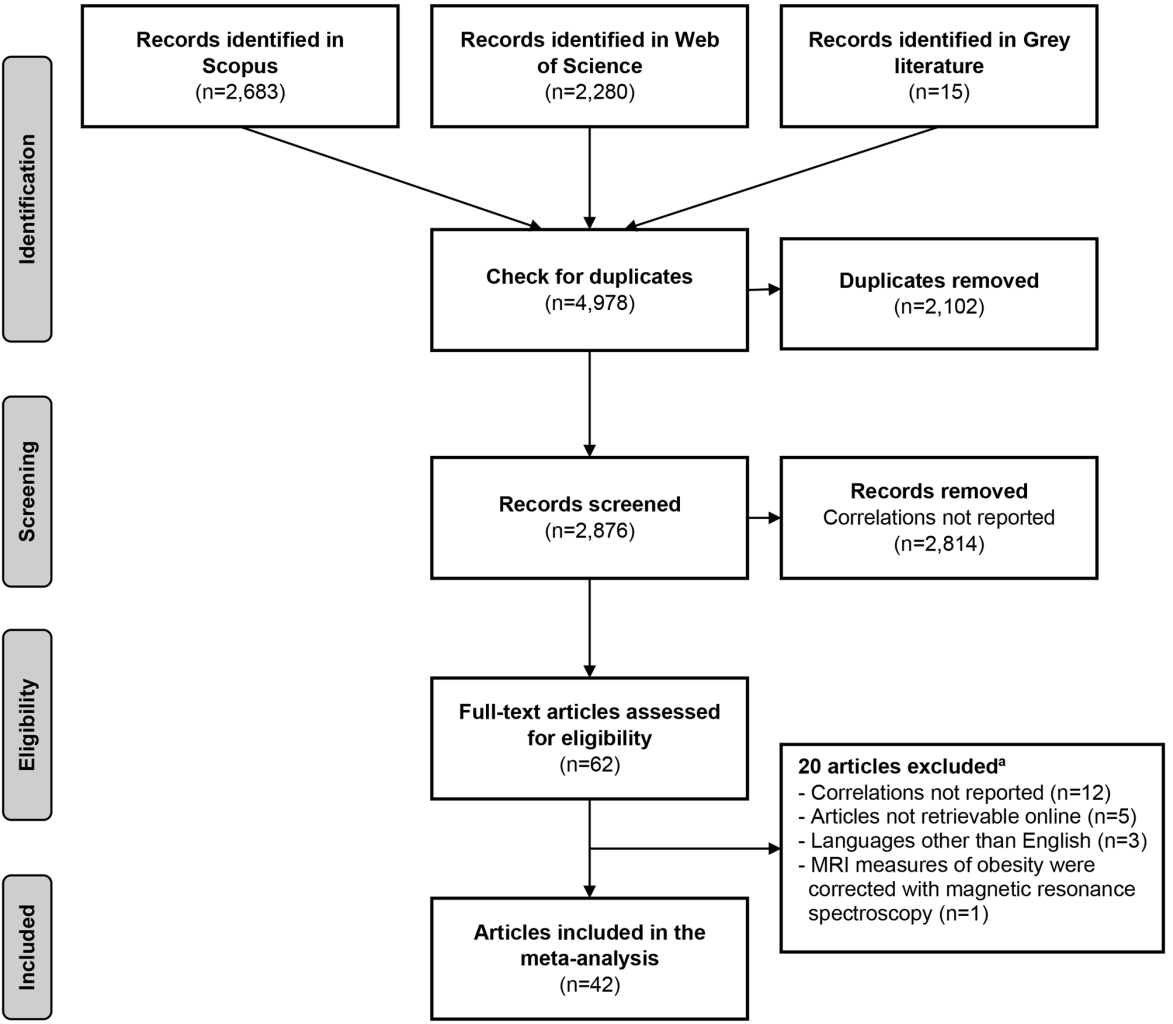
Flow diagram of selection procedures in the literature review. a. Details of the excluded studies can be found in Additional file 14

In total, the initial search identified 4,978 reports. After removing duplicate reports (n = 2,102), 42 studies were included in the meta-analysis, including 22 on MRI, 20 on DXA and 2 on both MRI and DXA (Fig. 1). Overall these 42 studies included a total of 42,556 participants, including 15,130 with information on MRI [32, 34–54] and 27,426 with information on DXA [32, 50, 55–72] (Table 1**)**. Participant characteristics of included articles in the literature review are presented in Additional files 3 and 4.

**Table 1:** Summary of participant’s characteristics in the meta-analyses

	Imaging method	
Characteristics	MRI	DXA
No. of studies	23^a^	20
No. of participants	15,130	27,426
Age group, %		
< 18 years	2.5	1.4
$\ge$18 years	97.5	98.6
Male,%	89.8	83.1
BMI range^b^, kg/m^2^	22.0–35.0	17.7–33.3
Ethnicity, %		
White	96.1	95.1
Non-White	3.9	4.9
Study setting, %		
Community	89.1	98.1
Hospital	8.5	1.5
Unspecified	2.4	0

a. The number of unique studies included was 22. The study by Kulberg et al., 2007 was included as two studies with distinct age groups (mixed younger adults and elderly adults)

b. mean or median according to what was reported in the study

Of the participants included, 98% were adults (i.e. 18 + years), 85% were men, 95% were White, and 95% were recruited from the general community. The mean or median BMI of study populations ranged from normal to obese class II according to the World Health Organisation criteria.

### Anthropometric vs. MRI-derived body composition

**Fig. 2:**
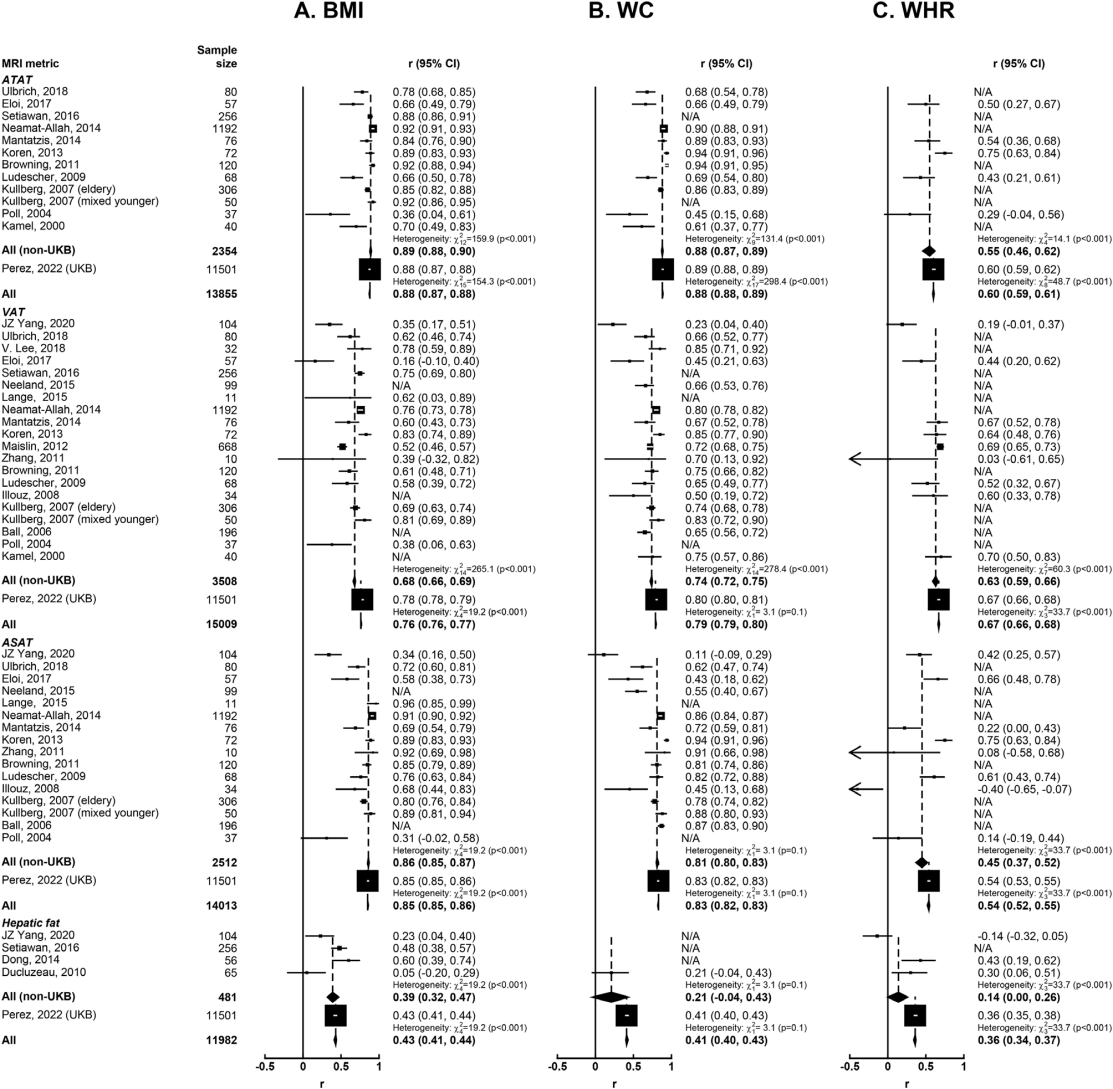
Correlations between MRI-derived adiposity and conventional anthropometric measures of adiposity. A black box denotes the correlation coefficient reported in each study with its size proportional to (n -3), where n is the sample size of the study. A diamond denotes the meta-analysed overall correlation coefficient with the solid line indicating zero correlation. Heterogeneity is assessed with a Q-test Abbreviations: MRI = magnetic resonance imaging; r = correlation coefficient; BMI = body mass index; WC = waist circumference; WHR = waist to hip ratio; ATAT = abdominal total adipose tissue; VAT = visceral adipose tissue; ASAT = abdominal subcutaneous adipose tissue; N/A = not available

Figure 2 presents the weighted average correlations between anthropometric and MRI measures of body fat composition. Overall, BMI and WC showed very strong correlations with ATAT (BMI: r = 0.88, 95%CI 0.87–0.88; WC: 0.88, 0.88–0.89) and ASAT (BMI: 0.85, 0.85–0.86; WC: 0.83, 0.82–0.83), and strong correlation with VAT (BMI: 0.76, 0.76–0.77; WC: 0.79, 0.79–0.80). Compared with BMI and WC, the corresponding correlations between WHR and MRI-derived measures of body fat composition were generally lower (ATAT: 0.60, 0.59–0.61; VAT: 0.67, 0.66–0.68; ASAT: 0.54, 0.52–0.55). Unlike BMI and WC, WHR was more strongly correlated with VAT than with ASAT. However,  BMI and WC still showed greater correlations with VAT than did WHR. All of the anthropometry measures were at best only weakly to moderately correlated with hepatic fat (BMI: 0.43, 0.41–0.44; WC: 0.41, 0.40–0.43; WHR: 0.36, 0.34–0.37). Overall, the results of the meta-analysis with and without the UKB study were comparable.

The correlations between anthropometric and specific MRI-derived measures of body fat varied to some extent by ancestry, sex, age and study setting. Notably, the correlations were significantly stronger in Whites than non-Whites for BMI with VAT (0.77 vs. 0.66) and ASAT (0.85 vs. 0.71);  for WC with VAT (0.80 vs. 0.58) and ASAT (0.83vs 0.69); and for WHR with VAT (0.67 vs. 0.36) (Additional file 5). Correlations were stronger in women than men for BMI with ATAT (0.92 vs. 0.88) and ASAT (0.91 vs. 0.85); but weaker in women than men for BMI with VAT (0.72 vs. 0.78), and WHR with ATAT (0.38 vs. 0.60); and VAT (0.55 vs. 0.67) (Additional file 6). There was also some evidence of differences by age, in that correlations were somewhat higher in those aged 18+  compared with those aged <18 for BMI with ATAT (0.88 vs. 0.82) and VAT (0.77 vs. 0.65); for WC with VAT (0.79 vs. 0.70), and for WHR with VAT (0.67 vs. 0.54). However, there was a lower correlation in those aged 18+ compared with those aged <18 for WHR with ASAT (0.54 vs. 0.69) (Additional file 7). There was some evidence that correlation coefficients between anthropometric and MRI-derived measures were somewhat greater in studies conducted within a community as opposed to a hospital setting. In particular, correlations were greater in community versus hospital based studies for BMI with VAT (0.78 vs. 0.59) and ASAT (0.86 vs. 0.61); for WC with ATAT (0.89 vs. 0.81), VAT (0.80 vs. 0.70) and ASAT (0.83 vs. 0.61); and for WHR with ATAT (0.60 vs. 0.47) and ASAT (0.54 vs. 0.06) (Additional file 8). At least some of the between study heterogeneity observed in certain pairwise comparisons (16 out of 28) may be due to differences in study populations according to one or more of ancestry, sex, age and study setting (Additional files 5–8).

### Anthropometric vs. DXA-derived body composition

**Fig. 3:**
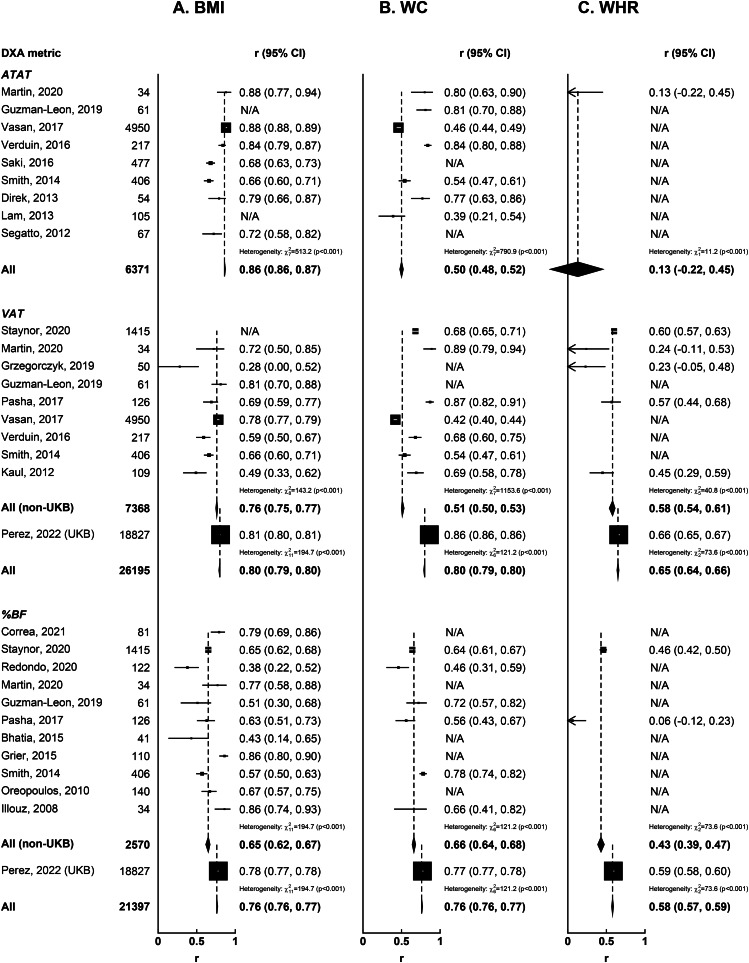
Correlations between DXA-derived measures of adiposity and conventional anthropometric measures of adiposity. Conventions as in Fig. 2. Abbreviations: DXA, dual x-ray absorptiometry; r, correlation coefficient; BMI, body mass index; WC, waist circumference; WHR, waist to hip ratio; ATAT, abdominal total adipose tissue; VAT, visceral adipose tissue; %BF, percent body fat; N/A, not available

Figure 3 shows correlations of anthropometric with DXA-based measures of body fat. BMI was most strongly correlated with ATAT (0.86, 0.86–0.87), followed by VAT (0.80, 0.79–0.80) and %BF (0.76, 0.76–0.77). Compared with BMI, the correlations between WC and DXA-derived measures of body fat were generally weaker for ATAT (0.50, 0.48–0.52), but not for VAT (0.80, 0.79–0.80) or %BF (0.76, 0.76–0.77). Likewise, compared with WC, WHR showed weaker correlations with ATAT (0.13, -0.22-0.45), VAT (0.65, 0.64–0.66) and %BF (0.58, 0.57–0.59), albeit based on relatively few studies. Overall, the results of the meta-analysis with and without the UKB study were comparable, but for correlation of WC with VAT the addition of the UKB study increased the correlation from moderate to strong (0.51 vs. 0.80).

Women showed stronger correlations than men for BMI with VAT (0.81 vs. 0.74), and %BF (0.77 vs. 0.66); and for WC with ATAT (0.61 vs. 0.40), and VAT (0.84 vs. 0.43) (Additional file 9). Correlations were significantly stronger in those aged 18+ compared with those <18 for BMI with VAT (0.80 vs. 0.59) and %BF (0.77 vs. 0.39); and for WC with VAT (0.80 vs. 0.68) and %BF (0.76 vs. 0.46), but weaker for WC with ATAT (0.48 vs. 0.84) (Additional file 10). At least some of the between study heterogeneity observed in certain pairwise comparisons (9 out of 14) may be due to differences in the study populations according to one or more of sex and age (Additional files 9 and 10). None of the studies included reported correlations of interest by ancestry groups, and subgroup analyses by study setting were not feasible for DXA related measures, because too few studies with DXA measures were conducted within a hospital setting (< 2%).

In general, correlations of all anthropometric measures with imaging-derived ATAT and VAT were higher for MRI- than DXA-derived measures, the only exception being for BMI and WC, which showed a slightly greater correlation with DXA- than MRI-derived VAT (Additional file 11).

### Publication bias

Although the number of included studies was relatively small, funnel plots showed evidence of an asymmetric distribution for some pairwise comparisons, with the majority of the smaller studies clustering to the left of the mean Fisher’s Z correlations, suggesting some degree of publication bias (Additional files 12 and 13). The formal tests showed nominally significant results for correlations of (a) BMI versus MRI-derived and DXA-derived VAT, (b) WC versus MRI-derived VAT, and (c) WHR versus DXA-derived VAT, which may be due to the different population characteristics between studies. For example, the MRI studies included a mixture of large and small studies, with sample sizes ranging from 10 to 11,501, and participants recruited from hospital and community settings, and at different ages.

## Discussion

This systematic literature review and meta-analysis of 42 studies provides a comprehensive summary of the available evidence regarding the correlation between imaging-based body fat composition and conventional anthropometric measurements. We found that both BMI and WC were very strongly correlated with MRI-derived total and subcutaneous fat in the abdominal area, and to a slightly lesser extent, with visceral abdominal fat. In contrast, WHR showed moderate to strong correlations with all MRI-derived measures, which were somewhat stronger with visceral than with subcutaneous abdominal fat. All the anthropometric measures considered were weakly to moderately correlated with MRI-derived hepatic fat. In general, correlations of anthropometric with imaging-based abdominal total fat and visceral abdominal fat tended to be higher for MRI-derived than for DXA-derived metrics. For certain pairwise comparisons, there was evidence of heterogeneity across certain population subgroups (e.g. somewhat stronger correlations in women than men for WC with ATAT and VAT, and in Whites). Although the UK Biobank study [32] accounted for around 85% of all male participants included in the meta-analyses, the overall correlation estimates were relatively similar with and without inclusion of this study, suggesting that the presence of high proportion of men did not unduly influence the overall estimates.

It remains unclear to what extent imaging-derived adiposity may improve our understanding of obesity-related diseases. In a few small cohort studies (sample size around 3,000) that have measured body fat using computed tomography, there was good evidence that increased VAT, hepatic fat and pericardial fat are associated with certain cardio-metabolic risk factors such as impaired glucose and hypertension [73–76], and cancer [77] after adjusting for BMI or WC. Some of these studies reported correlations ranging from weak to strong between BMI and visceral fat (-0.19-0.61), and between WC and visceral fat (0.23–0.66), and reported very weak correlations of BMI and WC with hepatic fat (-0.19 and -0.04, respectively) [73, 74].

A key finding from this study is that BMI, which is generally viewed as a measure of overall or general adiposity, had similar if not higher correlations with imaging-based measures of visceral fat as did WC and WHR, which are commonly used as anthropometric markers of abdominal and/or visceral fat. In fact, although the correlation of BMI with MRI-derived visceral fat (0.76, 0.76–0.77) was slightly smaller than that for WC (0.79, 0.79–0.80), it was slightly greater than that for WHR (0.67, 0.66–0.68). The more modest correlation of WHR with visceral fat compared to that of BMI or WC may reflect a greater degree of error in its measurement since, unlike BMI and WC, it is derived from two separate body measurements (waist and hip) [78–80]. Although anthropometric measures of adiposity are strongly correlated with visceral fat, they are not well correlated with measures of ectopic fat, as evidenced by their modest correlations with MRI-derived liver ectopic fat. It should be noted, however, that these correlations were mainly based on a mixture of direct and indirect comparisons of different participants. Future large studies of different measures of adiposity in the same study populations are needed to confirm (or refute) the present study findings.

Previous studies have suggested that both MRI and DXA can provide an extremely accurate assessment of adipose tissue distribution [36, 47, 81–85]. Unfortunately we were not able to directly assess this because none of the studies included in the present meta-analyses simultaneously applied these two imaging techniques to the same participant. Nevertheless, our indirect comparison of the data showed that the correlations of anthropometric with MRI-derived adiposity measures were consistently higher than corresponding correlations with DXA-derived measures. Differences in study population characteristics between studies reporting on MRI- and DXA- based measures could have contributed to the apparent differences in the agreement between anthropometric measures and DXA-derived compared with MRI-derived measures. In particularl the fact that significant differences were found in the agreement of the latter between community versus hospital based participants may support the above argument.

As in the present study, previous studies have reported modest differences in the correlation between imaging and anthropometric measurement by sex, ancestry and age [34, 38, 42, 44–47, 49, 50, 56, 57, 61, 63, 68, 70, 86]. There is also evidence that body fat composition differs by ancestry, with East and South Asians more likely to accumulate visceral fat, in comparison to other ancestry populations [87]. Although we were not able to investigate these ancestry groups, we found evidence of significant differences between Whites and non-Whites, albeit based on small number of participants, in correlations of WHR with various imaging-based adiposity measures. It is well established that body shape and fat distribution differ by sex, with men more likely to store fat around their abdomen, known as an android pattern, and women more likely to store subcutaneous fat around their hips, buttocks and thighs producing a body profile known as gynoid pattern [88]. These differences in fat distribution by sex may have contributed to the consistently higher correlations which we observed of BMI with abdominal total, subcutaneous and visceral fat in women than men. Ageing also affects body fat distribution with the amount of visceral fat increasing and subcutaneous fat decreasing with age [89]. We found some evidence of differences by age with the most notable those aged 18+ having a higher correlation than those <18  for all the anthropometric measures with both MRI- and DXA-derived visceral fat. It is well established that certain diseases (e.g. sarcopenia and cachexia) affect body fat distribution [90]. We found some evidence of greater correlations between anthropometric and MRI-derived adiposity measures in those studies that were community based. This may suggest that anthropometric indices are a poorer measure of specific body fat components in those with underlying health conditions. Previous studies also reported that activity levels, medications, dietary habits, alcohol and smoking consumption affect the body fat profile [91, 92], but it was not possible to investigate these since the correlations of interest were not available in the published studies.

This is the first systematic literature review and meta-analysis to investigate the correlations between imaging-derived and anthropometric measurements of adiposity, including more than 15,000 individuals with MRI-derived body fat measures, and more than 27,000 with DXA-derived measures. However, our study also had several limitations. Firstly, the included studies had inconsistent definitions of body fat components in the abdominal area. For example, one study defined visceral fat between L4 and L5 intervertebral disk [39], while another defined visceral fat between L1 and L5 [38]. Moreover, although most studies investigated total and/or subcutaneous abdominal fat, two studies [38, 42] measured these from head to toe and one study measured gluteal subcutaneous fat [93]. Furthermore, the imaging techniques and data extraction software used across the studies differed, particularly for MRI in terms of scanner brand, field strength, and segmentation method. Although such differences may not have materially affected the summary results, they could explain at least some of the heterogeneity observed between different studies. Secondly, although our meta-analyses included more than 40 individual studies, nearly all apart from the UKB study [32] included small sample sizes. Collectively, the total number of participants involved from these studies was not large and there was evidence of publication bias, which may lead to biased estimates and comparisons. On the other hand, it is possible that the skewed distribution in Funnel plots and Egger’s tests could also reflect heterogeneity between the studies due to different population characteristics. Thirdly, given the limited power, our subgroup findings related to ancestry, sex and age should be interpreted with caution and require further validation in larger studies of diverse populations. Fourth, there are other imaging-derived adiposity metrics (e.g. pericardial and epicardia ectopic fat, muscle fat infiltration, and pancreatic fat) that would be important to investigate in relation to anthropometric measures of adiposity, but these were unavailable in the published literature.

In summary this meta-analysis demonstrated that conventional anthropometric measures of adiposity, particularly BMI and WC, are strongly correlated with imaging-derived total abdominal, subcutaneous abdominal, and to a slightly lesser extent with visceral abdominal fat, but are weakly correlated with imaging-derived hepatic fat. Further studies involving simultaneous assessment of anthropometric and different imaging-based measures in large population-based studies are needed to further validate the present study findings, to extend the analyses to other imaging-derived measures of body fat and fat composition, and to further assess the relationship of different measures of adiposity with risks of specific diseases in diverse populations.

### Electronic supplementary material

Below is the link to the electronic supplementary material.

Supplementary Material 1

Supplementary Material 2

Supplementary Material 3

Supplementary Material 4

## Data Availability

All data generated or analysed during this study are included in the published articles listed in Additional files 3 and 4.

## List of abbreviations

ASAT: Abdominal subcutaneous adipose tissue
ATAT: Abdominal total adipose tissue
BMI: Body-mass index
BF: Body fat
CI: Confidence interval
DXA: Dual x-ray absorptiometry
MRI: Magnetic resonance imaging
r: Correlation coefficient
TAT: Total adipose tissue
VAT: Visceral adipose tissue
WC: Waist circumference
WHR: Waist-hip ratio
