# Supplementary material for: Assessment of correlation between conventional anthropometric and imaging-derived measures of body fat composition: a systematic literature review and meta-analysis of observational studies
Source: BMC Med Imaging. 2023 Sep 14;23:127. doi: 10.1186/s12880-023-01063-w (PMC10503139; doi:10.1186/s12880-023-01063-w)
Supplement: Supplementary file 1 — Supplementary Material 1 [file 12880_2023_1063_MOESM1_ESM.docx]

**Additional files**

[Additional file 1. Inclusion and exclusion criteria used in the literature search. 2](#_Toc125983776)

[Additional file 2. Search strategy used. 3](#_Toc125983777)

[Additional file 3. Characteristics of selected MRI studies included in the meta-analysis. 4](#_Toc125983778)

[Additional file 4. Characteristics of selected DXA studies included in the meta-analysis. 6](#_Toc125983779)

[Additional file 5. Correlations between MRI-derived and conventional anthropometric adiposity measures by ancestry. 9](#_Toc125983780)

[Additional file 6. Correlations between MRI-derived and conventional anthropometric adiposity measures by sex. 10](#_Toc125983781)

[Additional file 7. Correlations between MRI-derived and conventional adiposity measures by age. 11](#_Toc125983782)

[Additional file 8. Correlations between MRI-derived and conventional adiposity measures by study setting. 12](#_Toc125983783)

[Additional file 9. Correlations between DXA-derived and conventional adiposity measures by sex. 13](#_Toc125983784)

[Additional file 10. Correlations between DXA-derived and conventional adiposity measures by age. 14](#_Toc125983785)

[Additional file 11. Weighted average correlations between MRI-derived (black), DXA-derived (grey) and conventional measures of adiposity. 15](#_Toc125983786)

[Additional file 12. Funnel plots of study specific correlations between correlations of MRI-derived and anthropometric measures of adiposity. 16](#_Toc125983787)

[Additional file 13. Funnel plots of study specific correlations between DXA-derived and anthropometric measures of adiposity. 17](#_Toc125983788)

[Additional file 14. References of excluded studies. 18](#_Toc125983789)

Additional file 1. Inclusion and exclusion criteria used in the literature search.

| **Criteria** | **Inclusion** | **Exclusion** |
| --- | --- | --- |
| **Population** | Adults and/or adolescents | 1. Infants 2. Non-humans |
| **Outcome** | Correlation coefficient between any one of the specified anthropometric measures of adiposity (BMI, WC, WHR) and any one of the specified MRI-derived or DXA-derived adiposity measures (ATAT, VAT, ASAT, %BF) | MRI- derived adiposity corrected with magnetic resonance spectroscopy |
| **Date** | Between January 2000 and January 2023 | Before January 2000 and after January 2023 |
| **Language** | English | Manuscript unavailable online |

Additional file 2. Search strategy used.

| **Database** | **Search term** |
| --- | --- |
| **Scopus** | TITLE-ABS-KEY ( ( "D? XA"  OR  "Dual*energy X*ray absorptiometry"  OR  "MRI"  OR  "magnetic*resonance imaging" )  AND  ( "anthropometric*"  OR  "BMI"  OR  "body*mass*index"  OR  "weight*"  OR  "waist*circumference*" )  AND  ( "body composition"  OR  "fat distribution"  OR  "adipose tissue"  OR  "adipos?"  OR  "obesity" ) )  AND  PUBYEAR  >  1999  AND  ( LIMIT-TO ( LANGUAGE ,  "English" ) ) |
| **Web of Science (Editions: Science Citation Index Expanded; Social Sciences Citation Index; Arts & Humanities Citation Index; Conference Proceedings Citation Index- Science; Conference Proceedings Citation Index-Social Science & Humanities; Books Citation Index - Social Sciences & Humanities; Emerging Sources Citation Index; Current Chemical Reactions; Index Chemicus)** | ("D? XA" OR "Dual*energy X*ray absorptiometry" OR "MRI" OR "magnetic*resonance imaging") AND ("anthropometric*" OR "BMI" OR "body*mass*index" OR "weight*" OR "waist*circumference*") AND ("body composition" OR "fat distribution" OR "adipose tissue" OR "adipos?" OR "obesity") (All Fields) and English (Language) |

Additional file 3. Characteristics of selected MRI studies included in the meta-analysis.

| First author, publication year, country | Ethnicity, % | N | Males, % | Mean age^a^, years | Mean BMI^a,^ kg/m^2^ | Weight/height measured by trained examiner | Study settings | Adjustment for confounders |
| --- | --- | --- | --- | --- | --- | --- | --- | --- |
| Perez‑Cornago, 2022, UK | White 100 | 11501 | 100 | 57 | 29 | Yes | Healthy community | No |
| JZ Yang, 2020, New Zeeland | White 51,  Other 29, Asians 20 | 104 | 0 | 53 | 28.1 | Yes | Healthy community | No |
| Ulbrich, 2018, Switzerland | White | 80 | 0 | 21-62 | 17.5-26.2 | Yes | Healthy community | No |
| V. Lee, 2018, USA | White | 32 | 0 | 9.3-13.7 | 15.3-33.4 | Yes | Healthy community | No |
| Eloi, 2017, Brazil | White | 57 | 0 | 16-18 | 18-35 | Yes | Healthy community | No |
| Setiawan, 2016, USA | White 19,  Black 19, Native Hawaiian 21,  Japanese American 22, Latino 19 | 256 | 0 | 68.4 | 28 | Self-reported | Hepatocellular carcinoma | Age |
| Neeland, 2015, USA | White 80, Asian 18  Black 2 | 99 | 0 | 55.6 | 32.4 | N/A | Type 2 diabetes | No |
| Lange, 2015, Germany | White | 11 | 0 | 13 | 18-35 | Yes | Healthy community | No |
| Neamat-Allah, 2014, Germany | White | 1192 | 0 | 47-81 | 17-40.5 | Yes | Healthy community | Age, height |
| Mantatzis, 2014, Greece | White | 76 | 0 | 38-72 | 31.5 T2DM, 30.1 healthy | Yes | 50% T2DM, 50%no metabolic syndrome | No |
| Dong, 2014, China | Asian | 56 | 0 | 43-58 | <25 n=24, >=25 n=18 | Yes | n=42 healthy community, n=14 impaired glucose tolerance | No |
| Koren, 2013, USA | African-American 71 White 29 | 72 | 0 | 12-16 | 18-35 | Yes | Healthy community | No |
| Maislin, 2012, Iceland | White | 668 | 0 | 20.9-83.2 | 20-51.2 | Yes | Obstructive sleep apnoea | No |
| Zhang, 2011, China | Asian | 10 | 0 | 9-14 | 29 | Yes | Community-based obese children | No |
| Browning, 2011, UK | White | 120 | 0 | 18-79 | 27.5 men, 27.4 women | Yes | Healthy community | No |
| Ducluzeau, 2010, France | White | 65 | 71 | 57 | 31 | N/A | People with one or more CVD risk factor addressed by their general practitioner | No |
| Ludescher, 2009, Germany | White | 68 | 0 | 42.3 | 24.4 | N/A | n=39 healthy community, n=17 depressive syndrome, n=13 bulimia nervosa | Age, sex |
| Illouz, 2008, France | White | 34 | 74 | 59 | 33.2 | N/A | Obese, T2DM with metabolic syndrome | No |
| Kullberg, 2007, Sweeden | White | 306 | 52 | 70 | 27 men,  26.5 women | Yes | N/A | No |
| Kullberg, 2007, Sweeden | White | 50 | 54 | 14-66 | 26.8 men, 26.2 women | Yes | N/A | No |
| Ball, 2006, USA | Latino | 196 | 58 | 8-13 | >25 | Yes | BMI more or equal to the 85th percentile, Latino background positive family history for T2DM and absence of T2DM | No |
| Poll, 2004, Germany | White | 37 | 87 | 48 | 27.9 | Yes | Diabetes | No |
| Kamel et al, 2000, USA | White | 40 | 0 | 26-57 | 30-39.9 | Yes | Healthy community based | No |

a. Or shown as range of values using “-“, or otherwise stated.

Additional file 4. Characteristics of selected DXA studies included in the meta-analysis.

| First author, publication year, country | Ethnicity, % | N | Males, % | Mean age^a^, years | Mean BMI^a^, kg/m^2^ | Weight / height assessed by a trained examiner | Study settings | Adjustment for  confounders |
| --- | --- | --- | --- | --- | --- | --- | --- | --- |
| Perez‑Cornago, 2022, UK | White 100 | 18827 | 100 | 57 | 29 | Yes | Healthy community | No |
| Correa, 2021, Brazil | White 67, Black 12, Other 21 | 81 | 100 | 18-35 | 24.7 | Yes | Healthy community | No |
| Staynor, 2020, West Australia | White 82, Other 18 | 1,415 | 48 | 18-65 | 23.0 (21.1–25.6) | Yes | Healthy community | No |
| Redondo, 2020, USA | White 77, Hispanic 12, African-Americans 7, Other 4 | 122 | 32 | 12-19.5 | >25 | Yes | Type 1 diabetes | No |
| Martin, 2020, South Africa | Black | 34 | 62 | 43.9 | 25.7 | Yes | HIV positive undergoing haemodialysis | No |
| Grzegorczyk, 2019,  Poland | White | 50 | 0 | 51-85 | 28.5 | N/A | Healthy community based post-menopausal | No |
| Guzman-Leon, 2019, Mexico | Other | 61 | 52 | 20-37 | 24.7 | Yes | Healthy community based | No |
| Pasha, 2017, USA | White 57,  Asian 4 African-American 6,  Hispanic 5, Other 8 | 126 | 45 | 49.1 | 28 | Yes | Healthy community | No |
| Vasan, 2017, UK | White | 4,950 | 44 | 29-55 | 25.2 | Yes | Healthy community | BMI, fat mass index |
| Verduin, 2016,  Netherlands | White | 217 | 48 | 10-11 | 17.7 | Yes | Healthy community | No |
| Saki, 2016, Iran | Other | 477 | 51 | 89-19 | 17.7 | Yes | Healthy community | No |
| Bhatia, 2015, USA | White | 41 | 78 | 10-17 | 33.3 | Yes | Healthy community obese/overweight children with habitual snoring | No |
| Grier, 2015, USA | White 74, Asian 3, Black 9  Hispanic 9 | 110 | 100 | 23 | 26.4 | Yes | Army soldiers | No |
| Smith, 2014, Australia | White | 406 | 45 | 74-94 | 26.9 | N/A | Healthy community based without psychotic symptoms, schizophrenia, bipolar disorder, multiple sclerosis, motor neuron disease, developmental disability, or progressive malignancy, and dementia | No |
| Direk, 2013, UK | White | 54 | 0 | 49.3-72.8 | 25.1 | Yes | Healthy community | No |
| Lam, 2013, China | Asians | 105 | 52 | 21-65 | 28.1 | Yes | Healthy community | No |
| Kaul, 2012, USA | White 92, Hispanic 4, Asian 4 | 109 | 44 | 18-90 | 26.7 | Yes | Healthy community | No |
| Segatto, 2012, Brazil | White | 67 | 58 | 44 | 26.7 | Yes | HIV/AIDS | No |
| Oreopoulos, 2010, Canada | White 90 African American, Asian 6, Other 4 | 140 | 74 | 63 | 18-35 | N/A | Systolic and/or diastolic heart failure | Age and sex |
| Illouz, 2008, France | White | 34 | 74 | 59 | 33.2 | N/A | Obese, T2DM with metabolic syndrome | No |

a. Or shown as range using “-“, or median and (interquartile range), or otherwise stated.

Additional file 5. Correlations between MRI-derived and conventional anthropometric adiposity measures by ancestry.

Additional file 6. Correlations between MRI-derived and conventional anthropometric adiposity measures by sex.

Additional file 7. Correlations between MRI-derived and conventional adiposity measures by age.

Additional file 8. Correlations between MRI-derived and conventional adiposity measures by study setting.

Additional file 9. Correlations between DXA-derived and conventional adiposity measures by sex.

Additional file 10. Correlations between DXA-derived and conventional adiposity measures by age.

Additional file 11. Weighted average correlations between MRI-derived (black), DXA-derived (grey) and conventional measures of adiposity.

Additional file 12. Funnel plots of study specific correlations between correlations of MRI-derived and anthropometric measures of adiposity.

Additional file 13. Funnel plots of study specific correlations between DXA-derived and anthropometric measures of adiposity.

Additional file 14. References of excluded studies.

Reason for exclusion: Correlations not reported (1-12); articles not retrievable online (13-17); Languages other than English (18-20); MRI measures of obesity were corrected with magnetic resonance spectroscopy (7).

**References**

1. Pasanta D, Htun KT, Pan J, Tungjai M, Kaewjaeng S, Chancharunee S, et al. Waist Circumference and BMI Are Strongly Correlated with MRI-Derived Fat Compartments in Young Adults. Life-Basel. 2021;11(7).

2. Gadekar T, Dudeja P, Basu I, Vashisht S, Mukherji S. Correlation of visceral body fat with waist–hip ratio, waist circumference and body mass index in healthy adults: A cross sectional study. Medical Journal Armed Forces India. 2020;76(1):41-6.

3. Rowan JA, Rush EC, Plank LD, Lu J, Obolonkin V, Coat S, et al. Metformin in gestational diabetes: The offspring follow-up (MiG TOFU): Body composition and metabolic outcomes at 7-9 years of age. BMJ Open Diabetes Research and Care. 2018;6(1).

4. Lee V, Blew R, Hetherington-Rauth M, Blew D, Galons JP, Hagio T, et al. Estimation of visceral fat in 9- to 13-year-old girls using dual-energy X-ray absorptiometry (DXA) and anthropometry. Obesity Science and Practice. 2018;4(5):437-47.

5. Chen LW, Tint MT, Fortier MV, Aris IM, Bernard JY, Colega M, et al. Maternal macronutrient intake during pregnancy is associated with neonatal abdominal adiposity: The growing up in singapore towards healthy outcomes (GUSTO) study. Journal of Nutrition. 2016;146(8):1571-9.

6. Ablove T, Binkley N, Leadley S, Shelton J, Ablove R. Body mass index continues to accurately predict percent body fat as women age despite changes in muscle mass and height. Menopause-the Journal of the North American Menopause Society. 2015;22(7):727-30.

7. Sijens PE, Edens MA, Bakker SJL, Stolk RP. MRI-determined fat content of human liver, pancreas and kidney. World Journal of Gastroenterology. 2010;16(16):1993-8.

8. Schwenzer NF, MacHann J, Schraml C, Springer F, Ludescher B, Stefan N, et al. Quantitative analysis of adipose tissue in single transverse slices for estimation of volumes of relevant fat tissue compartments: A study in a large cohort of subjects at risk for type 2 diabetes by MRI with comparison to anthropometric data. Investigative Radiology. 2010;45(12):788-94.

9. Joy T, Kennedy BA, Al-Attar S, Rutt BK, Hegele RA. Predicting abdominal adipose tissue among women with familial partial lipodystrophy. Metabolism: Clinical and Experimental. 2009;58(6):828-34.

10. Bozkirli E, Ertorer ME, Bakiner O, Tutuncu NB, Demirag NG. The validity of the World Health Organisation's obesity body mass index criteria in a Turkish population: A hospital-based study. Asia Pacific Journal of Clinical Nutrition. 2007;16(3):443-7.

11. Janssen LGM, Nahon KJ, Bracké KFM, van den Broek D, Smit R, Sardjoe Mishre ASD, et al. Twelve weeks of exenatide treatment increases [18F]fluorodeoxyglucose uptake by brown adipose tissue without affecting oxidative resting energy expenditure in nondiabetic males. Metabolism: Clinical and Experimental. 2020;106.

12. Lei SF, Liu MY, Chen XD, Deng FY, Lv JH, Jian WX, et al. Relationship of total body fatness and five anthropometric indices in Chinese aged 20-40 years: Different effects of age and gender. European Journal of Clinical Nutrition. 2006;60(4):511-8.

13. Bakir MA, Al-Bachir MM, Hammad KB, Habil KM, Ahmad HH. The accuracy of currently used WHO’s body mass index cut-off points to measure overweight and obesity in Syrian women: A correlation study. Epidemiology Biostatistics and Public Health. 2016;13(3).

14. Zheng XF, Tang QY, Tao YX, Lu W, Cai W. Clinical value of methods for analyzing the abdominal fat levels of obese children and adolescents. Obesity and Metabolism. 2010;6(4):105-10.

15. Zhang H, Peng Y, Liu ZX, Li SL, Lv ZL, Tian LF, et al. MRI and 1H-MRS in evaluation of the effects of acupuncture therapy on abdominal fat and hepatic fat content in obese children. Chinese Journal of Medical Imaging Technology. 2010;26(11):2127-30.

16. Jia WP, Lu JX, Xiang KS, Bao YQ, Lu HJ, Chen L. Prediction of Abdominal Visceral Obesity from Body Mass Index, Waist Circumference and Waist-hip Ratio in Chinese Adults: Receiver Operating Characteristic Curves Analysis. Biomedical and Environmental Sciences. 2003;16(3):206-11.

17. Jia W, Lu J, Xiang K, Bao Y, Lu H, Chen L. Evaluation of abdominal visceral obesity from anthropometric parameters using receiver operating characteristic curves. Zhonghua liu xing bing xue za zhi = Zhonghua liuxingbingxue zazhi. 2002;23(1):20-3.

18. Garcia RL, Carrasco JOL, Garcia LEC, Orocio RN. Trending overweight and obesity in college football players in Mexico. Retos-Nuevas Tendencias En Educacion Fisica Deporte Y Recreacion. 2021(40):289-95.

19. García CS, Barrera F, Labbé P, Liberona J, Arrese M, Irarrázaval P, et al. Quantification of visceral adipose tissue using magnetic resonance imaging compared with anthropometry, in type 2 diabetic patients. Revista Medica de Chile. 2012;140(12):1535-43.

20. Gomes MA, Rech CR, Gomes MBA, dos Santos DL. Correlation between anthropometric indices and body fat distribution in elderly woman. Revista Brasileira de Cineantropometria e Desempenho Humano. 2006;8(3):16-22.
