# Supplementary material for: Assessment of correlation between conventional anthropometric and imaging-derived measures of body fat composition: a systematic literature review and meta-analysis of observational studies
Source: BMC Med Imaging. 2023 Sep 14;23:127. doi: 10.1186/s12880-023-01063-w (PMC10503139; doi:10.1186/s12880-023-01063-w)
Supplement: Supplementary file 2 — Supplementary Material 2 [file 12880_2023_1063_MOESM2_ESM.docx]

### Editor: Leila jahangiryleila@gmail.com

Submitted: **24 Dec 2022**

#### Comments to the author

This systematic literature review and meta-analysis aimed to investigate the correlation between conventional anthropometric and imaging-derived measures of body fat composition. Please, revise the manuscript according to the reviewers comments.
