# Supplementary material for: Assessment of correlation between conventional anthropometric and imaging-derived measures of body fat composition: a systematic literature review and meta-analysis of observational studies
Source: BMC Med Imaging. 2023 Sep 14;23:127. doi: 10.1186/s12880-023-01063-w (PMC10503139; doi:10.1186/s12880-023-01063-w)
Supplement: Supplementary file 3 — Supplementary Material 3 [file 12880_2023_1063_MOESM3_ESM.docx]

**Reviewer reports submitted for version 1**

**Reviewer 1: Leila Namvar**

Submitted: **30 Nov 2022**

Feedback for the author(s)

1. You have stated in the discussion section that: “It remains unclear to what extent imaging-derived adiposity may improve our understanding of obesity-related diseases”. In the included studies, have authors looked at comorbidities and the presence of chronic diseases among participants? It is mentioned mostly are free from these conditions. Looking at the correlation between these conditions and adiposity, can give a great insight into predicting the impact of adiposity on health outcome of the individuals in the long term. If not, this should clearly be stated in the inclusion criteria. In other words, the reason to purposefully choosing studies with relatively healthy participants should be highlighted. The phrase “relatively healthy” should also be defined. In the additional files, there are participants, with diabetes, HIV positive, and hemodialysis and this may cause confusion for some readers. 2. The authors have indicated that the included studies have recruited participants from different settings (e.g., hospital, community, etc.). Authors could have reported any possible correlation, if any, between the place of recruitment with the main objective. Please explain and elaborate if place of recruitment has any impact on adiposity measure? 3. The BMI range of the participants in the study (MRI and DXA), have reported to be between 17.7 to 35.0 (Table 1). The authors could elaborate on whether this has been chosen on purpose and any aim to consider the specific BMI range among the participants. Final comments: The manuscript is very well written which is the results of a valuable comprehensive literature review with extensive attention to details. I think for future studies, it would be interesting to conduct similar systematic review for specific age groups with attention to the comorbidities as a result of chronic diseases. Specifically, the conditions that you have discussed in the introduction (e.g., heart disease, colon cancer, etc.) that are closely related to the adiposity.

**Reviewer 2: Khemayanto Hidayat**

Submitted: **14 Dec 2022**

Feedback for the author(s)

The present study qualitatively and quantitatively assessed the correlation between conventional anthropometric and imaging-derived measures of body fatness. I have several comments: Title Please add the study design to the title (i.e., a systematic review and meta-analysis of observational studies). Introduction While the basic information on various body fatness measures has been adequately mentioned in the introduction, the rationale for performing a systematic review and meta-analysis is unclear. It would be clearer if the authors could explicitly mention the necessity, novelty, and potential contribution of the present study by highlighting the existing research gaps that lead to the objectives of the current study. Eligibility Criteria The inclusion and exclusion criteria were not clearly defined. Please use the PICOS framework to define inclusion and exclusion criteria and present these criteria according to the framework in a table. It has been almost a year since the authors performed the database searches (25 January 2022). Please update the searches to avoid missing relevant articles. Please provide a complete search strategy for each database that can be replicated as supplementary tables. Statistical analysis It would be interesting to assess whether the observed results differ according to whether or not mutual adjustment for other measures of body fatness was performed. Results Please provide references for the 20 excluded articles.
