# Supplementary material for: Assessment of correlation between conventional anthropometric and imaging-derived measures of body fat composition: a systematic literature review and meta-analysis of observational studies
Source: BMC Med Imaging. 2023 Sep 14;23:127. doi: 10.1186/s12880-023-01063-w (PMC10503139; doi:10.1186/s12880-023-01063-w)
Supplement: Supplementary file 4 — Supplementary Material 4 [file 12880_2023_1063_MOESM4_ESM.docx]

## Reviewer reports submitted for version 2

### Reviewer 1: Khemayanto Hidayat

Submitted: **13 Mar 2023**

#### Feedback for the author(s)

Thank you for addressing my comments. I have no further comments.
